# An evaluation of coupling coordination between sports industry and health service industry in China

**DOI:** 10.1371/journal.pone.0256333

**Published:** 2021-08-18

**Authors:** Jinfu Xu, Shaoxiong Yang, Yu Lin, Ruoyu Yang

**Affiliations:** 1 Sports Industry Development Research Center, Fujian Jiangxia University, Fuzhou, China; 2 School of Physical Education and Sport Science, Fujian Normal University, Fuzhou, China; 3 Department of Physical Health, Fujian Sports Vocational and Technical College, Fuzhou, China; 4 School of law, Civil Aviation University of China, Tianjin, China; Institute for Advanced Sustainability Studies, GERMANY

## Abstract

Depending on the strategy of "Healthy China", more and more people pay attention to health issues. The integration and development of sports industry and health service industry is an inevitable outcome of industrial transformation and upgrading and healthy life in the new era. Through constructing the evaluation index system of the coupling and coordination development degree between sports industry and health service industry, using entropy evaluation method and coupling and coordination degree model, this paper explore the comprehensive level and coupling and coordination development status of sports industry and health service industry in thirty-one provinces, municipality cities and autonomous regions of China from 2013 to 2017. The results of this paper show that the comprehensive China’s sports industry and health service industry both present an incremental development trend year by year, and are characterized by the distribution of "high in the east and low in the west" in space. The government’s policy support provides superior industrial supporting conditions for the development of sports industry. However, it is not conducive to the promotion of industrial economic benefits. In the health service industry, the rapid development of health insurance is beneficial to the integration of industrial resources and the perfection of industrial chain. Whereas as the core content of health service industry, health service has greater space for development; the coupling and coordination degree between the two industries rises from mild maladjustment to basic coordination, which is characterized by the distribution of "high in the east and low in the west" in space; among provinces, with Beijing, the Yangtze River Delta and Guangdong as the three development center points, it shows the spatial evolutionary process from "dispersion-type plaques" to "gathering type scattered surfaces".

## Introduction

In recent years, China’s economy has continued to develop at a high speed, and its economic aggregate has leapt to a leading position in the world. While China’s economy is developing by leaps and bounds, the aging of the society, the destruction of ecological environment, the constant change of life style and the increasing aggravating of medical burden with chronic diseases as its core have become the new subjects that Chinese society urgently needs to cope with [1]. In 2012, the State Council of China promulgated A Notice on the Issuance of the "Twelfth Five-year" Plan for the Development of Health Business, which first proposed the concept of the "health service industry", and in the same year, it further set forth the main contents that the "health service industry" contains in A Notice on the Issuance of the "Twelfth Five-year" Plan for the Development of Service Industry. In 2013, Several Opinions on Promoting the Development of Health Service Industry promulgated by the State Council (hereinafter referred to as "Document No. 40") clarified the main tasks and policy measures of the health service industry, and proposed the development of diversified health service combining sports fitness with medical care. By 2020, we’ll strive to basically establish a health service industry system covering the full life cycle, rich in connotation and reasonable in structure, and forge a batch of famous brands and healthy service industrial clusters with virtuous circle, so that the health service industry will become an important strength to promote sustainable development of economy and society [2]. In 2014, the State Council of China pointed out in the Several Opinions on Accelerating the Development of Sports Industry and Promoting Sports Consumption (hereinafter referred to as "Document No. 46") that by 2025, we’ll basically establish a sports industry system with reasonable layout, perfect functions and complete categories to promote the integration and development of the sports industry, enrich the content of the sports industry, drive the combination of recreation and sports, popularize "exercise prescriptions", and give full play to the positive role of physical exercise in respects such as disease prevention and cure, and health promotion, so that the sports industry will become an important strength to promote sustainable development of economy and society [3]. In 2015, the State Council promulgated the Guiding Opinions on Accelerating the Development of Consumer-oriented Service Industries and Promoting the Upgrading of Consumption Structure, which proposed to promote the combination of sports and recreation, and further encourage the integration and development of related industry status such as sports industry and health service, with emphasis on improving the health quality and level of the whole people [4]. Driven by a whole set of policies, in recent years, the market scale of the health service industry in China increased from 1.62 trillion yuan in 2013 to 5.4 trillion yuan in 2018, with a compound annual growth rate (CAGR) of 27.23%. The added value of the Chinese sports industry increased from 356.3 billion yuan in 2013 to 1,007.8 billion yuan in 2018, with a compound annual growth rate of 23.12%. Both of these two industries are showing a trend of high-speed growing, and are marching from high-speed development towards high-quality development.

Thus far, in the context of the improvement of people’s consumption level, the enhancement of health consciousness and the powerful policy guidance efforts, as the health service industry and sports industry stride forward to a high-quality development phase, these two industries are also urged to upgrade their quality and expand their production capacity to the new industrial status and modes. As a result, in the current circumstances, the integration and development of the health service industry and the sports industry, which both belong to one of five major happiness industries in China, is the inevitable outcome of upgrading industrial structure and creating a healthy life, and is the practice of the idea of sustainable development of economy and society as well. In the above background, it is necessary to clarify several questions: (1) What’s the current development situation and degree of integration of sports industry and health service industry? (2) What kind of index system is used to measure the degree of development and coupling coordination between sports industry and health service industry? (3) What are problems during the integration process, and what kind of development path should follow in the future?

## Literature review

The study of industrial convergence began in the late 1970s. Along with the continuous development of social economy, correlation research on industrial convergence has also been gradually concerned by scholars, while coupling analysis is widely used in this domain as a kind of research method. The term of coupling originated from physics, and was subsequently introduced into the economic and social domains, which was used for the analysis of the linkage relations and the status of integration and development among different industries, economy and environment. Research methods are mature, and the research results are relatively rich. For instance, the coupling and coordination between related industries and regional sustainable development [5,6], the coupling between industry development and ecological environment [7], coupling and coordination development of different industries [8,9], etc.

Since the 1990s, the research on the integration of the sports industry has attracted the attention of scholars, and they have had a heated discussion on the fusion issues of industries such as sports and culture, tourism and providing for the aged. Xie, et al. pointed out that the coupling system of sports industry and urban sustainable development will go through six phases from low-level coordination symbiosis to development collapse and disintegration through qualitatively analyzing the coupling evolution process between sports industry and urban development [10]. Yang, et al. constructed the evaluation index system of sports industry and regional sustainable development, and adopted the coupling and coordination degree model to qualitatively analyze the current situation of coupling and coordination between the two. The research results show that the coupling coordination degree of sports industry and regional sustainable development has been improved significantly. However, the spatial spillover effect needs to be strengthened [11]. On the basis of analyzing the coupling effect mechanism of sports and industry for retired elder support, Zhuo, et al. constructed an evaluation system for the coupling and coordination development of the sports industry and pension industry, and analyzed the coupling and coordination status of the sports industry and pension industry in eleven provinces and cities in China’s Yangtze River Economic Zone. The results indicate that the general level of the two industries has an obvious rising trend and higher correlation. In addition, the coupling and coordination relations between the two industries present an incremental development trend year by year [8]. By using methods such as literature review and logical reasoning, Tian analyzed the realistic background and effects of the coupling development of the sports industry and tourism industry [12]. Chen made an empirical analysis after making clear the mechanism of action of coupling coordination development between two industries in Hunan Province. The results showed an increasing trends year by year of degree of coupling coordination, however, it was still at a low level [13]. Xu, et al. further quantitatively analyzed the coupling development status and spatial differential features of the two industries by constructing the evaluation system of coupling and coordination between sports and tourism industry, and expanding study area to 31 provinces, municipalities directly under the central government and autonomous regions in China. It pointed out that the coupling and coordination development of sports and tourism industry is slow, and the overall level is relatively low. The coupling coordination degree of these two industries presents a positive aggregation characteristic in space [14]. Lan, et al. measured the symbiotic relationship after clarifying the coordination development relationship between above two industries, and it pointed out there was a long-term asymmetric naturalism between above two industries [15].

With the continuous advancement of policies, in recent years, some scholars have also paid attention to discuss the integration and development of the sports industry and the health service industry. Yang pointed out that with aggravation of the aging degree in China, so that the industrial convergence of the two major service industries of sports and providing for the aged, and health has become extremely important. Relevant industry status such as recreation and sports and health preserving, and sports and providing for the aged have developed rapidly [16]. On the basis of analyzing the concept connotation, development current situation and future trends of the two industries, Wei, et al. proposed a synergetic development mode combining value chain value-added with industrial edge intersections aiming at the correlation and similarity between the two [17]. After in-depth interpretation of "Document No. 40" and "Document No. 46", Cha, et al. noted that the fusion development of sports industry and health service industry has already been powerfully supported and guaranteed by related policies, and discussed the fusion development path of the two industries from the three levels of government, market and society [18]. Song, et al. believed that a large number of research results on the integration of the sports industry and health service industry at present provide solid theoretical support for the integration of single athletic sports into the health service industry. After combining the relevant research literature such as fitness Qigong and health service industry, they analyzed the foundations and conditions, and logic and interactive relations of fitness Qigong integration and promotion of health service industry, and proposed the implementation way of the integration of the two industries from the levels such as government guidance, association operation, diversified financing, condensing professional personnel and forging information service platform [19]. In addition to this, there are also a series of correlation research that has carried out in-depth analysis on the development of the two industries from respects such as separation industry and product grade, and found that there exists a complementary and cohesive relationship between the two industries, and the industrial attributes are similar and closely related, which has laid the foundations and conditions for the coordinated development of the both [20–22].

To sum up, relative to the rich research results of the integration and development of the sports industry and other industries, the existing research of the sports industry and health service industry places more emphasis on the current situation, mechanism, model, development path and other respects, while the quantitative research on the integration and development of the sports industry and health service industry is rarely seen. The phenomenon of industrial convergence is a two-way dynamic development process. If we want to analyze it deeply, we need to study it by excavating data, establishing relevant index system and building a model [23]. Therefore, in order to comprehensively explore the current situation of the integration of sports industry and health service industry in China, on the basis of the existing relevant theoretical research, combining the panel data of the sports industry related index from 31 provinces, municipalities directly under the central government and autonomous regions (not including Hongkong, Macau, Taiwan) in China. we establish the evaluation index system of the coupling development of these two industries, construct the coupling and coordination degree model, measuring the comprehensive level and situation of coupling coordination of the sports industry and health service industry, to explore the distribution characteristics in space, to find out the disadvantage as of developments of above two industries, so as to provides useful reference for further promoting the integration and development of sports industry and health service industry.

## Research methods

### Entropy evaluation method

Considering that the traditional subjective weighting method has some limitations in calculating the index weight. Therefore, in order to abide by the objective and scientific principles, we calculate the index weight by adopting the entropy evaluation method. Entropy evaluation method empowerment can determine the weight with the numerical significance in accordance with the variability of indexes, and is not influenced by whether the evaluation data is linear or not. The assignment procedure is transparent and reproducible, and the index weight has higher reliability [8]. Therefore, this research calculates the index weight by adopting the entropy evaluation method, and then obtains the comprehensive development level index of sports industry and health service industry. Specific steps are as follows:

The first step: Since the dimensions and magnitude of different indexes have certain differences, in order to guarantee the accuracy of the analysis results, we need to standardize and make indexes being dimensionless of each index data first [24]. The standardized formula is $R_{ij}^{+}\;=\;\frac{x_{ij}-x_{j}^{min}}{x_{j}^{max}-x_{j}^{min}}$, In the formula, *x*_*ij*_ is the actual value of the index *j* in the year of *i*. $x_{j}^{max}、x_{j}^{min}$ represent the maximum and minimum value of the index *j* respectively. $R_{ij}^{+}\;$ represents the standardized data of the index in the year of *i*.
The second step: In order to eliminate the phenomenon that the result is 0 after standardized processing, we carry out translation processing on the standardized data. The translation processing formula is:$R_{ij}\;=\;H+R_{ij}^{+}$. Among which, the translation amplitude is *H*, and its value is 1.
The third step: We adopt the specific gravity method, and carry on the dimensionless to the index data. The dimensionless formula is $y_{ij}\;=\;\frac{R_{ij}}{\sum_{i\;=\;1}^{n}R_{ij}}$.
The fourth step: The entropy calculation formula of index *j* is $e_{j}=-\frac{1}{lnn}\sum_{i=1}^{n}y_{ij}\;ln\;y_{ij}$, and the difference coefficient calculation formula is *g*_*j*_ = 1- *e*_*j*_.
The fifth step: The weight calculation formula of index *j* is $\omega_{j}=\frac{g_{j}}{\sum_{j=1}^{n}g_{j}}$, among which, *j =* 1,2⋯ ⋯*n*, and *n* is the numbers of the indexes.
The sixth step: We calculate the comprehensive index of the coupling and coordination development degree between sports industry and health service industry. The formula is $Z_{i}=\sum_{j=1}^{p}\omega_{j}R_{ij}$. The comprehensive index can reflect the comprehensive level of the industry it represents. Namely, the higher the index, the higher the development degree. On the contrary, the lower the index, the lower the development degree [11].

### Coupling and coordination degree model

On the basis of the existing research, we can know that the degree of association between the sports industry and the health service industry is closely related. Therefore, we can discuss the current situation of mutual integration of the two by using the theory of coupling coordination. Coupling coordination degree can fully reflect the relationship between the coordinated development and virtuous circle of the two [25]. The calculation formula of coupling coordination degree is $D\;=\;\sqrt{C\times M}$. Among them, the specific calculation formulas of *C* and *M* are as follows:

$C={\left[\frac{Z_{s}\times Z_{h}}{{\left(Z_{s}+Z_{h}\right)}^{2}}\right]}^{\frac{1}{2}}$, *Z*_*s*_ is the comprehensive indexes of the sports industry, *Z*_*h*_ is the comprehensive index of the health service industry.

*M* = *αZ*_*s*_ + *βZ*_*h*_, *α* and *β* are undetermined coefficients. In consideration of the role of sports industry and health service industry in the course of coupling and coordination, and the value of *α* and β that Ye and He et al. assign when carrying on a research of the coupling coordination degree between sports industry and health service industry [8,26], while referring to the score results of five experts in related fields, we assign *α* and *β* as 0.5.

Finally, referring to the literature of two scholars Yang, et al. [11] and Zhuo, et al. [8], we classify those indicators, as shown in Table 1.

**Table 1:** The grade division of coupling coordination degree.

Coupling coordination degree (D)	Grade of coupling coordination degree	Stages
(0,0.200)	Serious maladjustment	Embryonic stage
(0.201,0.400)	Moderate maladjustment	Embryonic stage
(0.401,0.500)	Mild maladjustment	Embryonic stage
(0.501,0.600)	Basic coordination	Starting stage
(0.601,0.800)	Good coordination	Stable stage
(0.801,1.000)	High quality coordination	Mature stage

### Calculation method of Global Moran’I

This paper calculates the general levels of sports industry and health service industry, and the spatial association patterns of coupling and coordination degree between the two industries by adopting the value of Global Moran’I. The specific calculation formula is $I\;=\;\frac{\sum_{i\;=\;1}^{n}\sum_{j\neq 1}^{n}W_{ij}(x_{i}-\overset{-}{x})(x_{j}-\overset{-}{x})}{S^{2}\sum_{j\;=\;1}^{n}\sum_{j\;=\;1}^{n}W_{ij}}$. In the formula, n is the total number of areas, and *W*_*ij*_ is the space weight (when the area *i* and the area j is adjacent, *W*_*ij*_ = 1; on the contrary, *W*_*ij*_ = 0). *x*_*i*_ and *x*_*j*_ are the observed value of the area *i* and the area *j*. $\overset{-}{x}\;$ is the average value of *x*_*i*_, and *S*^2^ is the variance of *x*_*i*_. When the value of Global Moran’I is greater than 0 or less than 0, it illustrates that there is a positive or negative spatial agglomeration phenomenon; when the value of Global Moran’I is close to 0, it illustrates that there is no spatial association and it is a random distribution phenomenon [27].

## Evaluation index system construction and data sources

Because the interaction relationship between sports industry and health service industry is relatively complex in each factor, according to the integration mechanism of the two and on the basis of following the scientific, feasibility, systematicness and comprehensiveness principle of index selection, referring to the research results of Yang, et al. [11], Xu, et al. [13], Zhuo, et al. [8], Wei, et al. [15], He, et al. [26], and the formulation of the development system of sports industry and health service industry in relevant documents and policies, we determine the rough draft of the index system of this research. In order to further optimize the index system, we adopted the experts consultation method. After consulting seven experts in related fields, we adjusted the rough draft of the index system, and finally determined the index system of this study. Among them, the index system of the sports industry is consisted of eleven indexes, which includes three dimensions of economic benefit, employment effect and industrial support. The index system of health service industry consists of nine indexes, including three dimensions of health care, health service and health insurance. All indexes are positive. See Table 2 for detailed indicators. (The calculation method of indicator weight in Chapter 2.1 in the paper).

**Table 2:** The evaluation index system of coupling and coordination between the sports industry and the health service industry.

System layers	Subsystem layers	Indicator layers	Index weight
Sports industry	Economic benefit	The income from the main business of the sports goods manufacturing industry	0.107
		The operating revenue of sports service industry units	0.061
		The sales volume of sports lotteries	0.113
		Social fixed assets investment of sports enterprises	0.071
	Employment effect	Personnel who work in the sports goods manufacturing industry	0.062
		Personnel who work in the sports service industry	0.128
		Average wage of employed persons in sports enterprises	0.087
	Industrial support	Expenditures from government-managed funds (public welfare funds from lotteries)	0.104
		Institution numbers of sports cause units	0.114
		Local finance expenditures on sports	0.043
		Unit numbers of sports enterprises	0.109
Health service industry	Health care	Unit numbers of pharmaceutical enterprises	0.106
		Numbers of medical and health institutions	0.170
		Numbers of health technical personnel per 10,000 population	0.163
	Health service	Numbers of people of the proportion of outpatient service health examinations in medical and health institutions	0.028
		Investment in environmental pollution control as a proportion of GDP	0.093
		Unit numbers of health care services	0.098
	Health insurance	Numbers of people of the proportion of urban workers to participate in the endowment insurance	0.090
		Density of personal insurance	0.063
		Numbers of insured persons of the proportion of urban basic medical insurance at the end of the year	0.188

All the index data derives from the statistical yearbooks issued by the National Bureau of Statistics and relevant government departments, mainly including the *China Statistical Yearbook*, *China Statistical Yearbook of the Tertiary Industry*, *Sports Cause Statistical Yearbook*, *China Industry Statistical Yearbook*, *China Basic Unit Statistical Yearbook*, *China Statistical Yearbook on Environment*, and China Statistical Data Application Supporting System.

## The empirical analysis on the coupling development of sports industry and health service industry

### The analysis of comprehensive sports industry and health service industry levels

Based on the entropy evaluation method, we can calculate the comprehensive index of sports industry and health service industry in thirty-one provinces and cities in China. It can be seen from the data in Table 3 that, at the overall level of China, between 2013 to 2017, the comprehensive index of China’s sports industry and health service industry presents an incremental development trend year by year, and the comprehensive index of health service industry is higher than that of sports industry, which indicate that the comprehensive level of sports industry and health service industry has steadily improved within five years, and China as a whole belongs to the lagging type of sports industry. It is noteworthy that the compound annual growth rate of the comprehensive index of sports industry is 13.16%, and that of the comprehensive index of health service industry is 9.03%. From this, it can be illustrated that though the comprehensive level of the sports industry is lower than that of the health service industry, the comprehensive sports industry level is developing faster than that of the health service industry. Through further calculating the difference coefficients of comprehensive indexes of the two industries in thirty-one provinces and cities, we can find that the difference coefficients of the comprehensive indexes of the sports industry and the health service industry are 0.162 and 0.115 respectively, illustrating that the regional difference of comprehensive level of the sports industry is greater than that of the health service industry. The sports industry should lay emphasis on balanced development among provinces.

**Table 3:** Comprehensive index of sports industry, health service industry and various subsystems (year 2013–2017).

System (subsystem)	Nationwide							Eastern region		Central region		Western region	
	2013	2014	2015	2016	2017	Average	growth rate	Average	growth rate	Average	growth rate	Average	growth rate
Sports industry	0.179	0.216	0.241	0.267	0.294	0.24	13.16%	0.37	11.41%	0.217	14.26%	0.135	16.61%
Economic benefit	0.047	0.058	0.068	0.074	0.074	0.064	12.21%	0.11	8.97%	0.057	16.53%	0.027	19.62%
Employment effect	0.051	0.061	0.068	0.077	0.086	0.069	13.81%	0.104	9.29%	0.059	16.36%	0.043	23.00%
Industry support	0.082	0.096	0.105	0.116	0.134	0.107	13.28%	0.156	14.57%	0.101	12.00%	0.065	11.85%
Health service industry	0.247	0.255	0.257	0.279	0.349	0.277	9.03%	0.336	7.25%	0.254	11.95%	0.24	9.11%
Health care	0.108	0.11	0.113	0.116	0.119	0.113	2.42%	0.116	2.73%	0.125	3.07%	0.103	1.56%
Health service	0.048	0.044	0.041	0.047	0.051	0.046	1.49%	0.052	5.14%	0.038	0.48%	0.046	-1.59%
Health insurance	0.091	0.101	0.104	0.116	0.179	0.118	18.49%	0.167	11.27%	0.09	28.66%	0.092	22.86%

Note: The eastern region includes Beijing, Tianjin, Hebei, Liaoning, Shanghai, Zhejiang, Fujian, Shandong, Guangdong, Hainan; the central region includes Shanxi, Jilin, Heilongjiang, Anhui, Jiangxi, Henan, Hubei, Hunan; the western region includes Inner Mongolia and Guangxi, Chongqing, Sichuan, Guizhou, Yunnan, Tibet, Shaanxi, Gansu, Qinghai, Ningxia, Xinjiang.

From the comprehensive indexes of the three subsystems of the sports industry, we can find that the comprehensive level of industrial support is the highest, while the comprehensive level of economic benefits and the compound annual growth rate are the lowest. The results are basically consistent with those of Yang, et al. [11]. Since the promulgation of the "Document No. 46", the State Council has also successively issued a series of relevant policies, starting out with encouraging social capital to enter the field of the sports industry, setting up special funds for the sports industry, increasing government procurement service, implementing preferential policies on taxes and dues, increasing financial support strength and other respects, paying dividends at the policy level, brushing aside the development obstacles for the sports industry, with the aim of stimulating the market vitality of the sports industry and the people’s enthusiasm for consumption, providing powerful support for the sustainable and high-quality development of the sports industry, and driving the sports industry to become a pillar industry of the national economy. Therefore, depending on government departments to provide powerful policy support for the sustainable and high-quality development of the sports industry, the comprehensive level of industrial support is obviously higher than the employment effect and economic benefits.

In terms of economic benefits, though the added value of China’s sports industry has presented rapid growth in recent years, the added value of the sports industry accounts for 1.1% of GDP (Gross Domestic Product), and the role of the sports industry in fueling economic growth has begun to take shape. However, the data in Table 3 shows that the comprehensive level of economic benefits is significantly lower than that of employment effect and industrial support. On the one hand, this phenomenon shows that there is still a larger room for improvement in the economic benefits of the sports industry; on the other hand, it shows that our country should fully excavate and release the role of market subjects in the sports industry, so as to stimulate the economic benefits of the sports industry. At present, in the course of the development of China’s sports industry, the government is still in a leading role, which reduces the enthusiasm of a lot of social capital integration. A lot of enterprises and social organizations themselves lack development orientation and incentive mechanism, thus resulting in their lack of momentum. At the same time, due to the existing problems in the links such as the local government’s investment in the support funds for the sports industry, evaluation management mechanism, financial aid forms and later-period management, it will result in risks such as market order destruction, pulling efficiency loss and project performance missing [28], making a lot of enterprises rely on acquiring the government’s various kinds of support funds as their basic means of survival. This phenomenon has influenced the development of the comprehensive level of economic benefits of the sports industry to a great degree.

In the health service industry system, the comprehensive level basis of health care is superior to that of health service and health insurance. However, health insurance grew at a compound annual growth rate of 18.49%, showing a rapid development momentum, and exceeded the comprehensive level of health care for the first time in 2017. As the sunrise industry, the health service industry had the phenomenon of excessive medicalization in the early phase of its development [29]. With the continuous maturity of industry development, on the basis of traditional medical knowledge and technique, the health service industry has gradually evolved into a new type of health service industry to meet the ever-increasing requirement of the masses for health service. Therefore, driven by the traditional medical industry, the comprehensive level of health care has a relatively solid foundation. Health insurance has the role of resources integration in the health service industry chain, which is the entrance of people’s health service demand, and is conducive to the promotion and perfection of the health service chain. From the *Notice on Relevant Matters regarding the Provision of Health Management Services by Health Insurance Products* issued by China Insurance Regulatory Commission in 2012 to the *Measures for Administration of Health Insurance* newly revised by the State Council in 2017, they both mentioned supporting commercial insurance companies to innovate health insurance products, expand the cooperation with institutions such as medical treatment, physical examination and nursing, and construct the health service industry chain. In recent years, while improving the coverage rate of basic medical insurance in China, commercial health insurance has also made considerable development. In 2018, the original premium of China’s commercial health insurance increased to 544.813 billion yuan, with the growth rate ranking the first amongst all types of insurance. At the same time, the research on the demand degree and satisfaction degree for the health insurance service industry of 1,187 residents in thirty-four cities in China shows that after the health service industry is subdivided into eleven industries, the residents’ demand degree and satisfaction degree for the health insurance service industry are both higher [30]. Therefore, under the support of national policies, driven by urbanization, and under the condition of the improvement of people’s requirements for a healthy life, a good political, economic and social environment has been created for the development of comprehensive health insurance level.

The comprehensive level and compound annual growth rate of health services are both inferior to those of health care and health insurance. The reasons that cause this phenomenon are mainly the following in aspects: Firstly, the understanding of "service" is not thorough enough. The emphasis of the health service industry rests with he word "service". At the present stage, however, the connotation understanding of the word "service", the humanistic basis, and the construction of the material base are all still in the development phase from all sectors of society in China, and there is a short of standardization of health service informatization [31]. Secondly, there is a shortage of professional talents. Due to the late start of related education in the health service industry, it results in a serious gap of compound talents in medical and health management. However, as the most important core element of enterprise and social development, the deficiency of talents will inevitably result in backward technical services. Thirdly, the proportion of health service industry is the smallest. As the core content of the health service industry, the relevant data shows that the proportion of the health service industry in the whole health service industry is only 2.71%, which is far from other industries. Therefore, in order to further improve the level of China’s comprehensive health service industry, it is absolutely imperative that we need to put forth effort on perfecting the current situation of health service development, that is, clarifying the position and development ideas of health service, cultivating and condensing the professional talent team, relaxing the market access system of health management and health service industry, and perfecting the regulations and standards of health services, so as to create a good social atmosphere for the health service industry.

According to the data in Table 3, from the sub-regional level, it is observed that the comprehensive sports industry and health service industry levels in China are characterized by the ladder-like distribution of the highest level in the east, the second level in the middle, and the lowest level in the west in space. Among them, the eastern region belongs to the retarded type of health service industry, while the central and western regions belong to the retarded type of sports industry. The distribution characteristics are influenced by factors such as social and economic strength, infrastructure conditions, scientific and technological education level, talent resources, and degree of opening to the outside world to a great degree [27]. The compound annual growth rate of the comprehensive level of sports industry is characterized by the distribution of “the highest in the western part, the second in the middle part, and the lowest in the eastern part”, which illustrates that the central and western regions, which originally had a weak foundation of sports industry, give play to the superiority conditions such as regional geographic characteristics and resource endowment, and motivate theinternal potential of the sports industry with the boost of favorable policies such as the "rising strategy in central region", "sports aid for Tibet" and "the development of the western region in China", thus urging the rapid development of the comprehensive level of sports industry. The compound annual growth rate of the health service industry is manifested as the central part is higher than the western part, and the eastern part is the lowest. The central region of China, east to the coast, west to the inland, with convenient transportation and a solid foundation of agro-industry, has many national top 100 pharmaceutical enterprises and national first country-level medicine high-tech developmental zone such as Yangtze River Pharmaceutical Group and JUMPCAN; at the same time, Taizhou, Jiangsu Province commits itself to forging a massive health industry cluster district in the central region. As a result, under the guarantee of excellent geographical position, featured advantageous industries and high-efficiency system and mechanism, the growth rate of the comprehensive level of health service industry in the central region is higher than that in the eastern and western regions. It is noteworthy that the level of comprehensive health service in the western region is manifested as negative growth. Therefore, in the follow-up development course, on the basis of coordinating the overall development of health service, we should attach greater importance to the health service talents and technology in the western region and implement the policy inclination to guarantee the balanced development of the internal structure of the health service industry.

**Fig 1:**
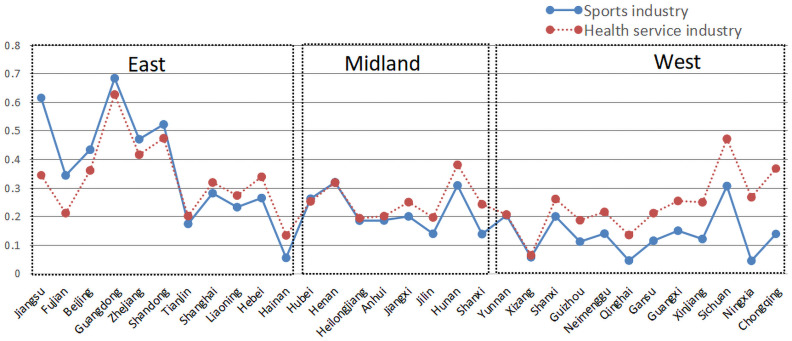
The development situation of comprehensive level of sports industry and health service industry.

In order to further explore the development situation of the comprehensive sports industry and health service industry levels in thirty-one provinces and cities in China, we selected the mean value of comprehensive index of the two industries in each province and city within five years for analysis. As can be seen from Fig 1, the top five provinces and cities with comprehensive sports industry level are from the eastern region, including Guangdong, Jiangsu, Shandong, Zhejiang and Beijing. Thus we can see that the provinces and cities with high-level development of our country’s sports industry are mainly concentrated in the eastern coastal areas, and through calculating, we find that the Global Moran’I value of comprehensive index of the sports industry is 0.2023, and passes the significance test at the 5% statistical level, further illustrating that the comprehensive sports industry level in thirty-one provinces and cities has positive agglomeration phenomenon in space. The top five provinces and cities in the comprehensive health service industry level, covering the eastern, central and western regions, are Guangdong, Shandong, Sichuan, Zhejiang and Hunan. The Global Moran’I value of comprehensive index of the health service industry is -0.0218, which fails to pass the significance level test, illustrating that the comprehensive health service industry level presents a random distribution characteristic in space.

At the time of investigating whether the comprehensive level development of the sports industry and health service industry is synchronous, when *Z*_s_ = *Z*_*h*_, it illustrates that the two industries belong to the synchronous development type; when *Z*_s_ > *Z*_*h*_, it is the retarded type of health service industry; when *Z*_s_ < *Z*_*h*_, it is the retarded type of sports industry [32]. As can be seen from Fig 1, except Jiangsu, Fujian, Beijing, Guangdong, Zhejiang and Shandong in the eastern region belong to the retarded type of health service industry, and Hubei and Henan in the central region belong to the synchronous development type, other provinces and cities belong to the retarded type of sports industry. The number of provinces and cities with the retarded type of sports industry is more than that of the retarded type of health service industry, which explains again that the comprehensive level of health service industry is superior to that of the sports industry. At the same time, the gap between the comprehensive level of the two industries in each province and city in the western region is obviously larger than that in the eastern and central regions. Whether this phenomenon illustrates that the degree of coupling and coordination development of the sports industry and health service industry in the western region is inferior to that in the eastern and central regions, this research will verify it through the rest of analysis.

### The analysis of coupling and coordination degree of sports industry and health service industry

The coupling and coordination degree is established on the basis of the comprehensive index and coupling degree, which can accurately reflect the coordinated development relationship between the sports industry and the health service industry. From the view of national level, the coupling and coordination degree of the sports industry and health service industry has expanded from 0.435 in 2013 to 0.544 in 2017, and the grade of coupling and coordination degree has changed from mild maladjustment to basic coordination; its development situation of coupling and coordination can be divided into two stages: The embryonic stage in 2013–2015, and the starting stage in 2016–2017, which shows that the two industries are gradually strengthening their ties, and the coupling and coordination relationship is developing in a benign direction.

**Fig 2:**
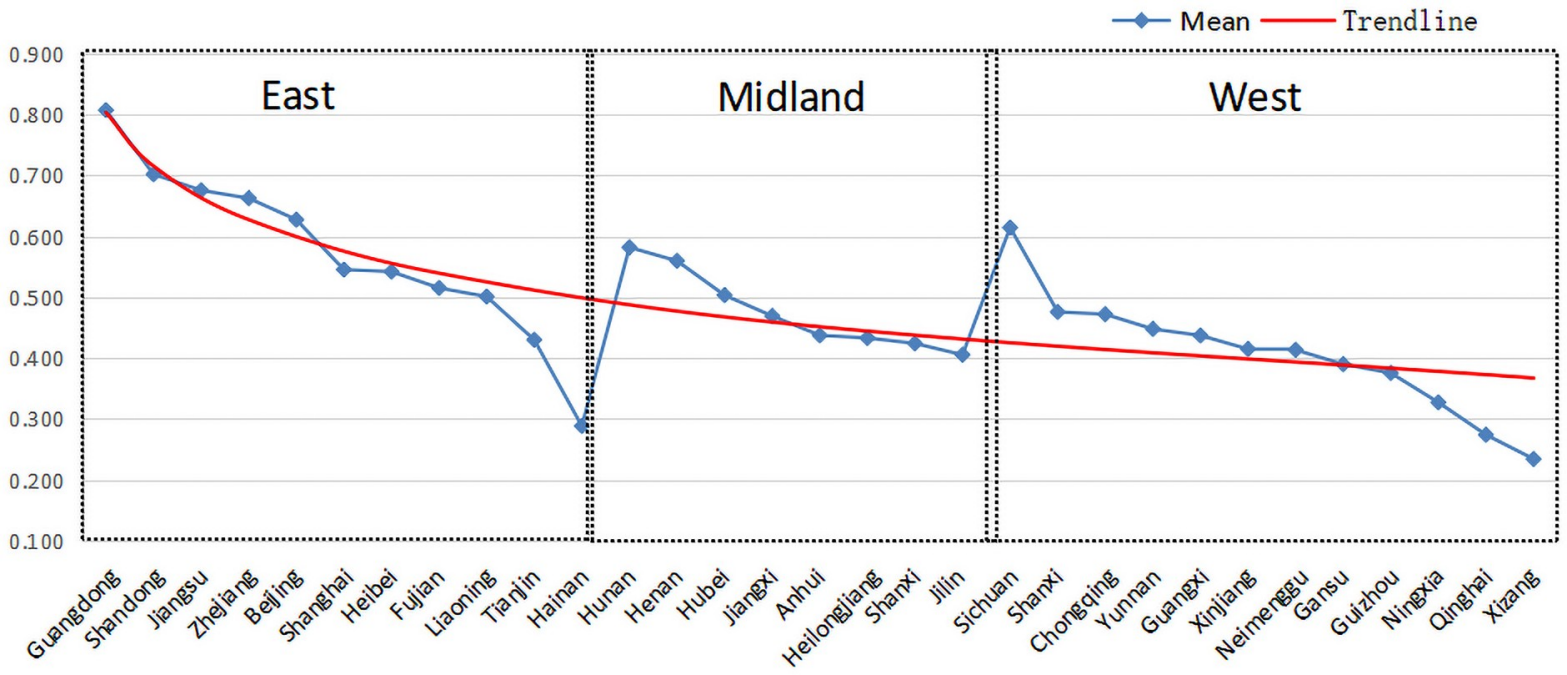
The mean value of index of coupling coordination degree of 31 provinces and cities in China within five years.

Fig 2 shows the mean value of index of coupling and coordination degree in thirty-one provinces and cities within five years. From the view of provincial condition, six provinces and cities of Guangdong, Shandong, Jiangsu, Zhejiang, Beijing and Sichuan have a higher degree of coupling and coordination, which belongs to the stage of stable maturity. Among them, Guangdong belongs to high-quality coordination, and the rest of five provinces and cities are good coordination. Guangdong, as the province with the largest economic scale, the strongest comprehensive competitiveness of economy and financial strength in China, is in the national leading position in the general sports industry and health service industry levels. Therefore, whether it is the external environment or the industrial self-development, are very beneficial to the integration and coordination of the two industries. Jiangsu, Zhejiang and Shandong, as strong sports provinces, sports industry has always kept a good development trend. At the same time, they strengthen the top-level design from the government level, optimize the development environment, lay emphasis on the cross-border integration of sports and health service industries, encourage merger and reorganization of enterprises and cultivate emerging health industries, thus achieving the upgrade of value chains. As the host places of the 2008 Summer Olympic Games and the 2022 Beijing Winter Olympic Games, Beijing has a good foundation for the sports industry development and presents a high-quality development trend; as one of the "high-grade precision and advanced" industries with the most innovation-driven characteristics, the most development strengths and the most in line with the requirements of high-quality development in Beijing, the health service industry is actively building two industry cluster districts of "basic research and development in the north and high-end manufacturing in the south"; in addition to this, in order to propel the accelerated fusion development of the sports industry and the health service industry, government departments have conducted strategic planning for the full integration of the sports industry into the health service industry from the respects of "sports medical rehabilitation industry" and "fitness and leisure sports industry". As a strong province in the health service industry of "Made in Sichuan" pharmaceutical boutiques with a national higher market share and a good brand reputation, Sichuan vigorously promotes the deep integration of national fitness and national health under the concept of "sports for use, health for the soul, and consumption as the foundation", and helps the two industries efficiently integrate.

From the trend line, it is observed that the coupling and coordination degree presents the characteristics of high in the east and low in the west. Concretely, a total of thirteen provinces and cities throughout the country have reached the starting stage, among which a total of nine are in the eastern region, accounting for 81.82% of the total in the eastern region; three in the central region, accounting for 37.50% of the total in the central region; and one in the western region, accounting for 8.33% of the total in the western region. Therefore, on the whole, The trends of coupling coordination degree and comprehensive development level are identical between sports industry and health service industry, presenting a distribution characteristic of "high in the east and low in the west". On the one hand, this phenomenon is influenced by the general sports industry and health service industry levels in various areas, on the other hand, it is due to the fact that the east region of China is superior to the central and western regions in respects such as economy, resources, living conditions and material base at present.

**Fig 3:**
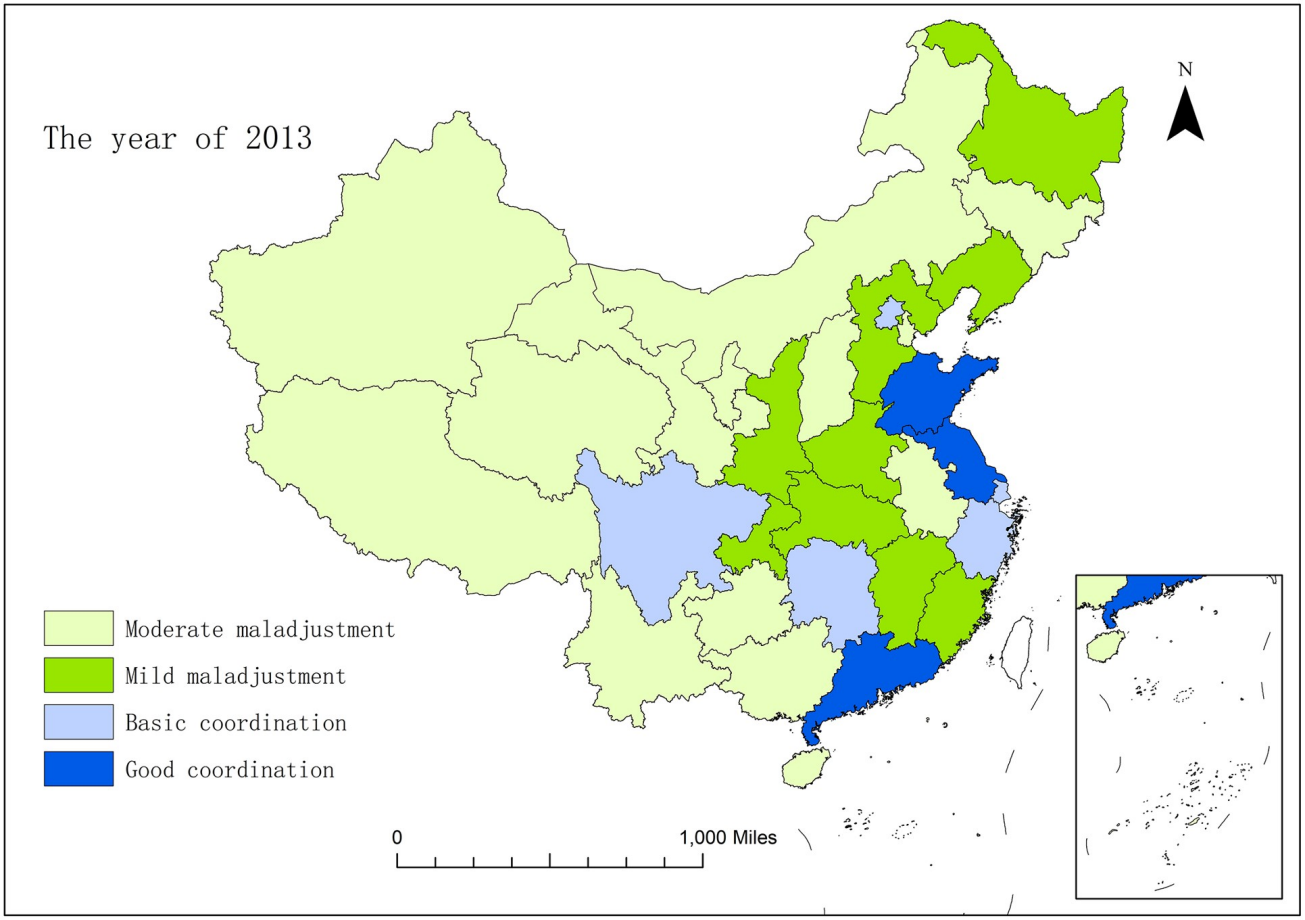
The spatial pattern of coupling and coordination of the sports industry and health service industry in 31 provinces and cities of China in the year of 2013.

**Fig 4:**
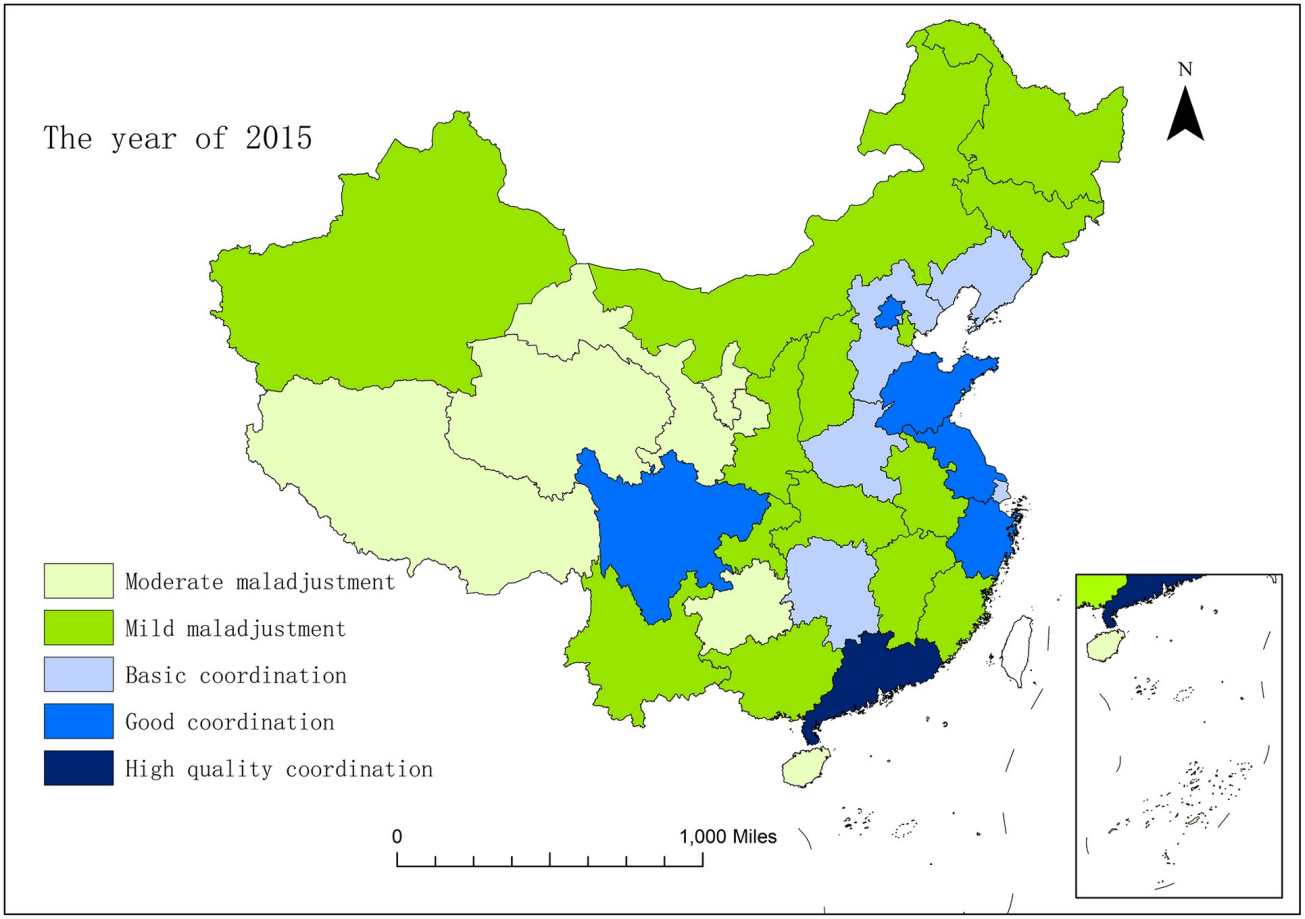
The spatial pattern of coupling and coordination of the sports industry and health service industry in 31 provinces and cities of China in the year of 2015.

**Fig 5:**
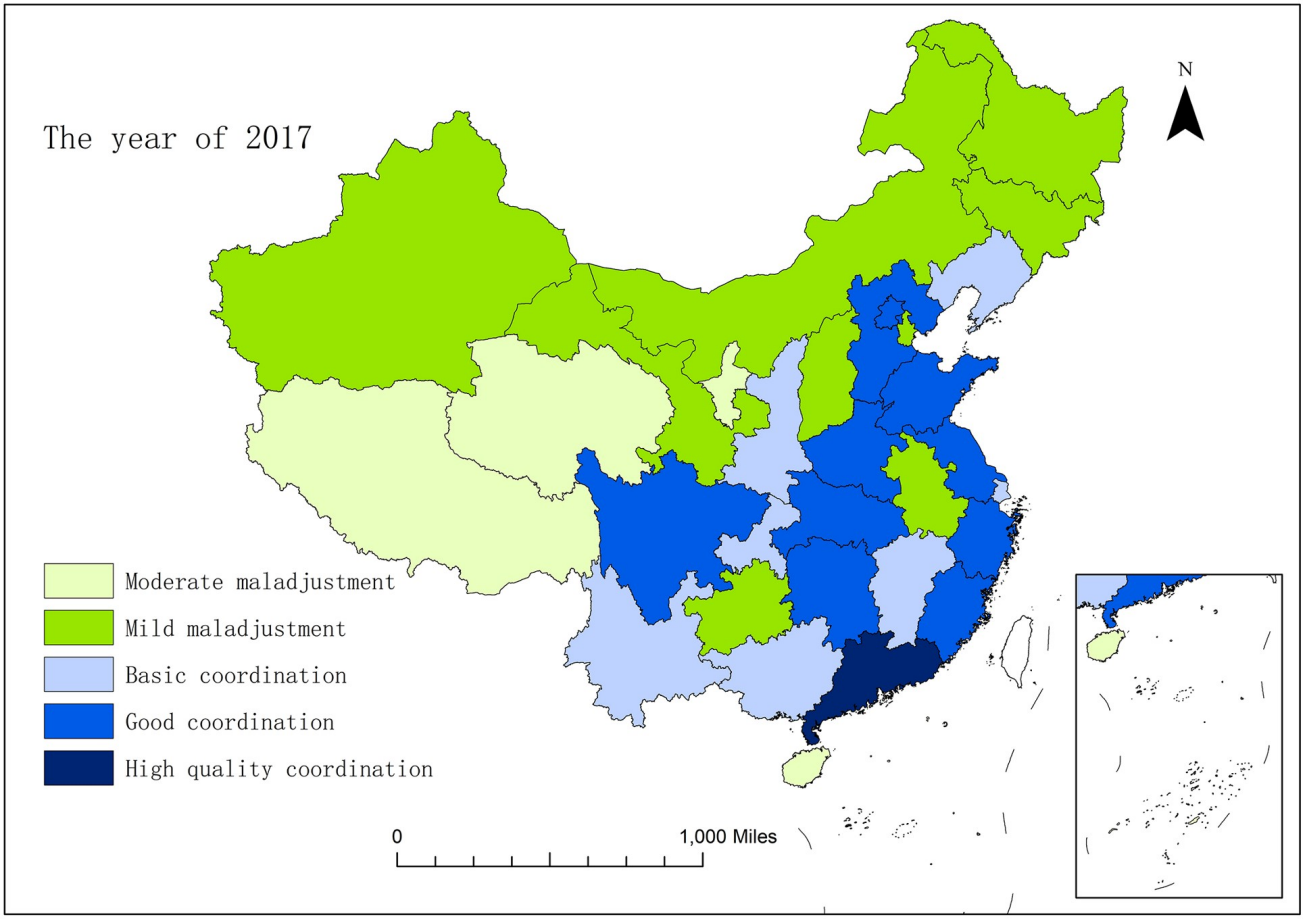
The spatial pattern of coupling and coordination of the sports industry and health service industry in 31 provinces and cities of China in the year of 2017.

In order to horizontally compare the development situation of coupling and coordination of the sports industry and health service industry in thirty-one provinces and cities in China, with the help of ArcGIS10.2 software, this study conducted spatial visualization of the coupling and coordination degree of the two industries between each province and city in 2013, 2015 and 2017. From Figs 3–5, we can intuitively find that in 2013–2017, the spatial pattern of regions above the starting stage changed from "dispersion-type plaques" in 2013 to "gathering type scattered surfaces" in 2017. The level of coupling and coordination development between the two industries in a majority of provinces and cities has been improved obviously.

In 2013, most provinces and cities throughout the country were in the embryonic stage, and the provinces and cities in the northwest region were all in the moderate maladjustment grade. Only Zhejiang, Shandong and Guangdong were in the stable stage, while Shanghai, Hunan and Sichuan were in the starting stage. By 2015, Guangdong, which was originally in the stable stage, had become the province that first strode forward the mature stage in China, with the coupling and coordination degree between the two industries being significantly improved. The three provinces and cities of Beijing, Fujian and Sichuan have also evolved from the starting stage to the stable stage, and have presented the situation taking Beijing as the center, and driving the surrounding provinces and cities to promote the coupling and coordination degree. Liaoning, Hebei and Henan evolved from mild maladjustment to basic coordination, and entered the starting stage from the embryonic stage. At the same time, the scope of provinces and cities in the northwest region which are in moderate maladjustment has been narrowed, and the coupling and coordination condition has been improved. In 2017, the coupling and coordination development of the two industries obtained remarkable effects. Beijing continues to play the role of the radiation effect of center point, and drives the further development of surrounding provinces and cities. Yangtze River Delta (Zhejiang, Shanghai and Jiangsu) and Guangdong, as two regions with a better foundation for coupling and coordination development, have become new development centre points, and begun to give play to the influential effect of spatial spillover. Therefore, from 2013 to 2017, the coupling and coordination development of the two industries were concretely manifested as gradually extent from the eastern coast to the inland with Beijing, the Yangtze River Delta, and Guangdong as the three centre points and the Changjiang River as the axis. This development also abides by the important law of world economic development of "starting and going ahead of the rest with the coast, and shaping the gradient development of inland rivers to depth hinterland".

**Fig 6:**
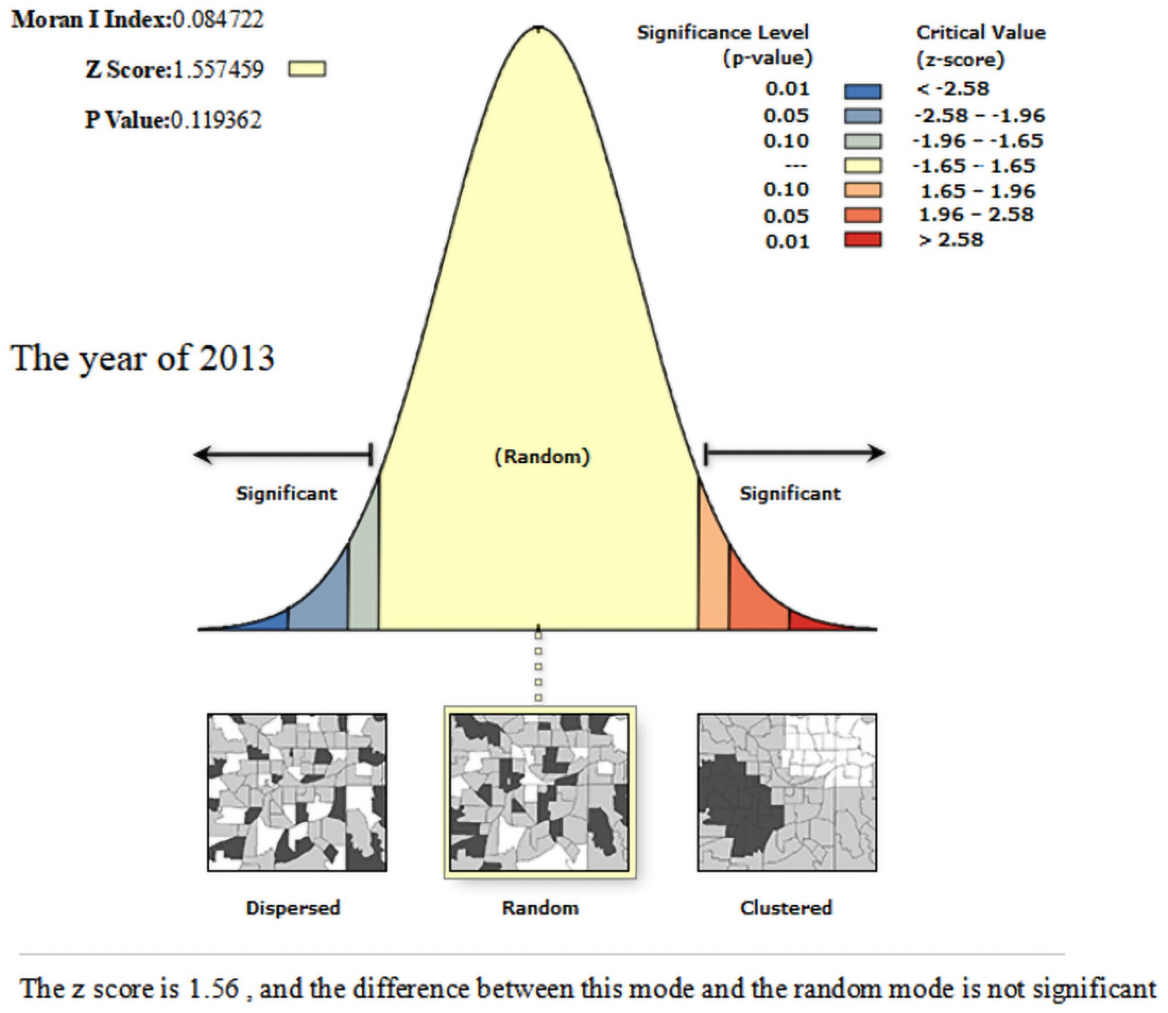
Spatial autocorrelation report of the coordination of the sports industry and health service industry of China in the year of 2013.

**Fig 7:**
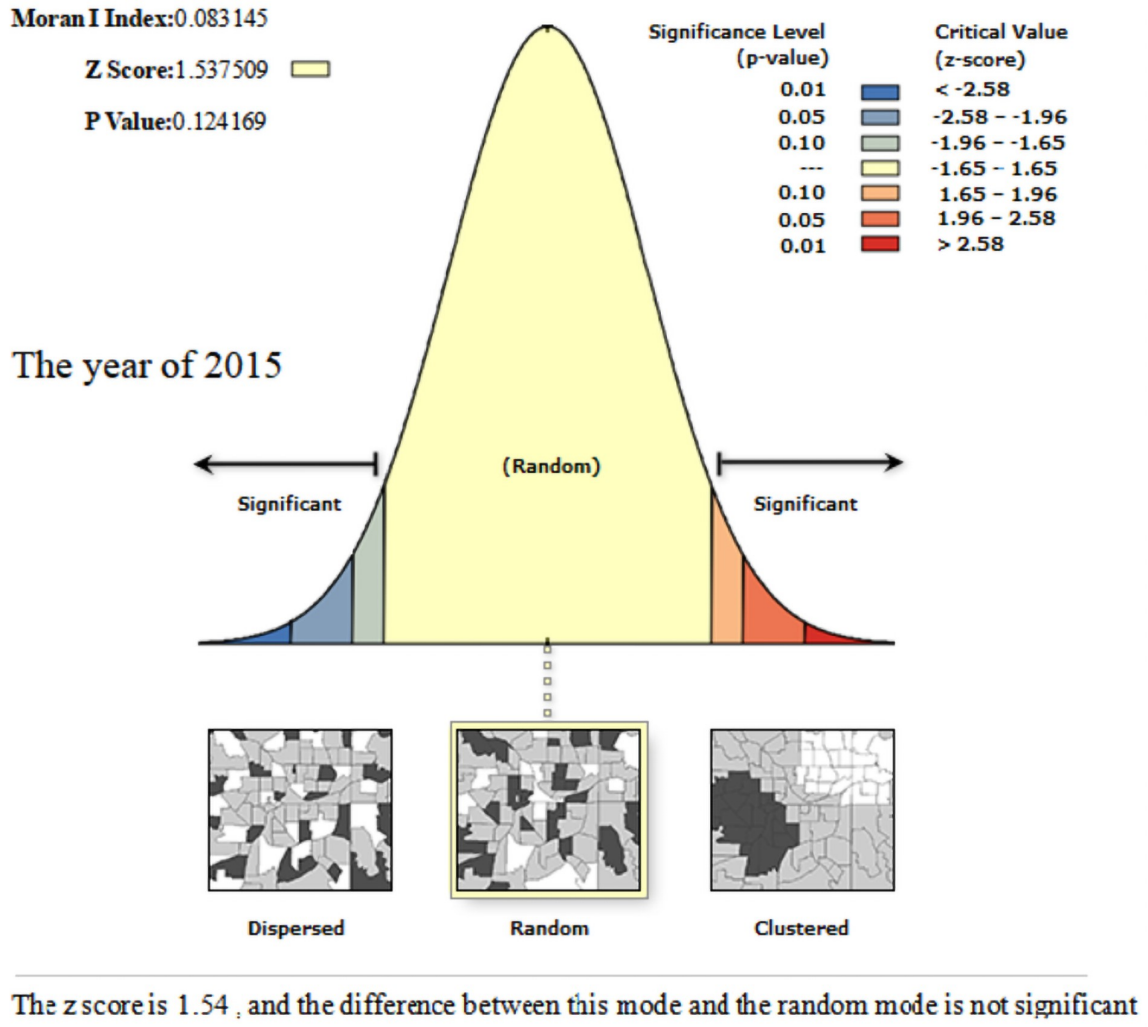
Spatial autocorrelation report of the coordination of the sports industry and health service industry of China in the year of 2015.

**Fig 8:**
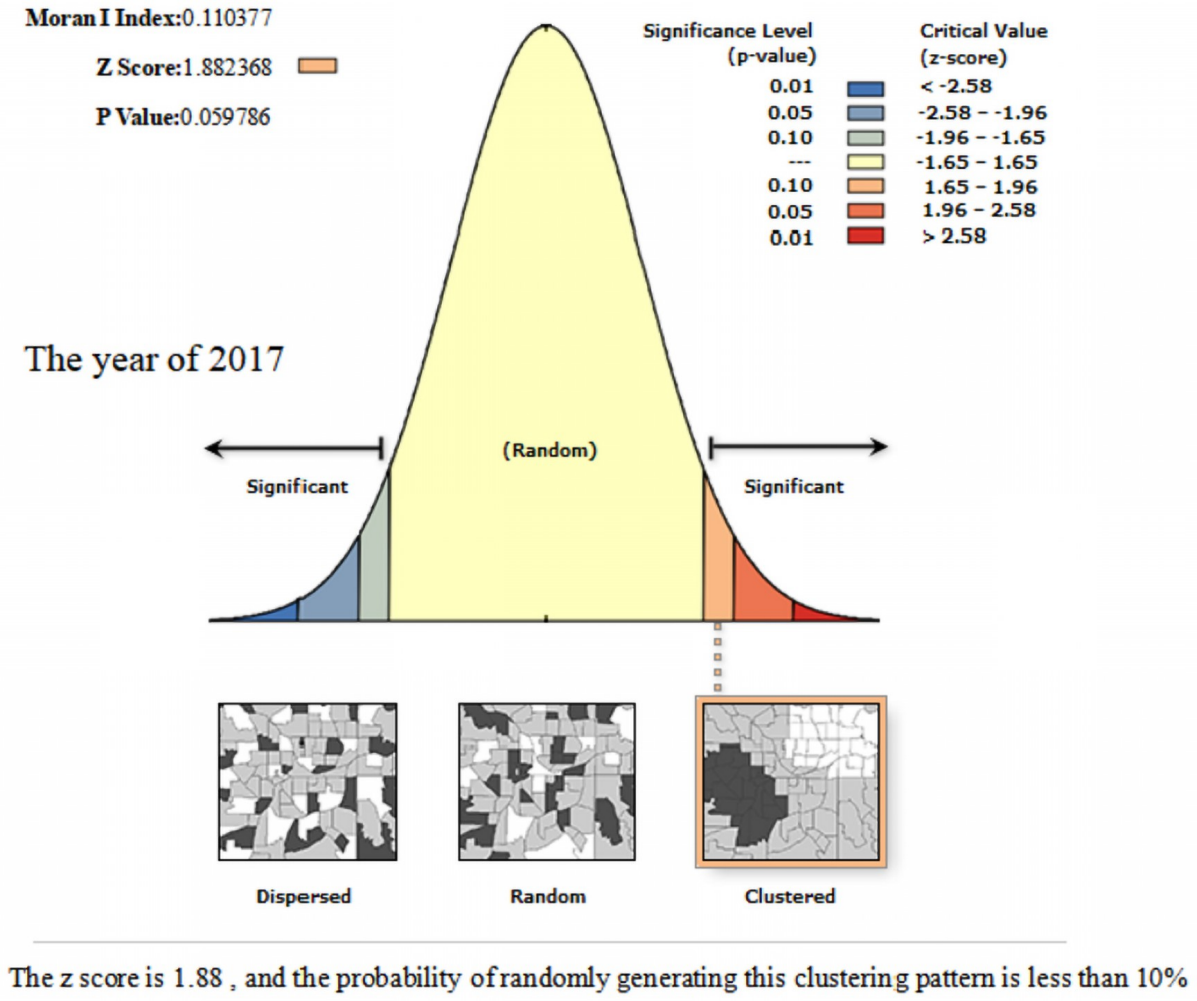
Spatial autocorrelation report of the coordination of the sports industry and health service industry of China in the year of 2017.

In order to further reveal the spatial association between the coupling and coordination development of the sports industry and the health service industry in thirty-one provinces and cities in China, we use ArcGIS10.2 software to calculate the *Global Moran’I* value of coupling coordination degree. From Figs 6–8, we can know that the *Global Moran’I* value in 2013–2015 was close to 0, and failed to pass the significance test of statistical level; the *Global Moran’I* value in 2017 was 0.1104, which passed the significance test at the 10% statistical level, illustrating that the coupling and coordination development of each province and city was random distribution in space in 2013–2015, and there was no aggregation phenomenon; with the influential action of the three centre point regions emerging, the coupling and coordination level of the surrounding provinces and cities in the centre point regions is improved, and the spatial spillover effect among the provinces is obvious. Therefore, it represents the spatial agglomeration characteristic of "gathering type scattered surfaces" in 2017.

## Conclusions

With the transformation of China’s economy in the new era and the in-depth development of the "Healthy China" strategy, people’s diversified requirements for sports and health are confronted with obvious supply side short plank, which has brought major development opportunities to the sports industry and health service industry, and also provided a moment for the integration and development of the two industries. Therefore, to explore the current situation of integration and development of the two is of great significance to facilitate the transformation and upgrading and coordinated development of the two industries. Through constructing the evaluation index system of coupling and coordination between the sports industry and the health service industry, this study deeply analyzes the comprehensive level and coupling and coordination status of the sports industry and the health service industry at the overall level, sub-regional level and provincial level in China by applying entropy evaluation method and coupling and coordination degree model. The results of research show that:

In 2013–2017, the comprehensive China’s sports industry and health service industry levels presented an incremental development trend year by year. The comprehensive health service industry level is superior to that of the sports industry. China’s overall performance is the retarded type of the sports industry. The comprehensive sports industry level is developing faster than that of the health service industry. However, the gap between provinces is more obvious than that of the health service industry as well.

In the development process of sports industry, the government’s powerful macro-control, while promoting the sports industry to support the development of the comprehensive level, also leads to the excessive dependence of some enterprises on the government, thus lowering the enthusiasm of market subjects. Therefore, the development of a comprehensive level of economic benefits of the sports industry is influenced. During the study period, it manifests that the comprehensive industrial support level is higher than that of economic benefit and employment support, and the comprehensive economic benefit level and the compound annual growth rate are lower than that of industrial support and employment effect.

In the health service industry, benefiting from the solid foundation that the traditional medical industry provides, in the early phase, the comprehensive health care level is higher than that of health services and health insurance. With the improvement of people’s living standards, the increase of per capita disposable income, and the mature development of the health service industry, the comprehensive health insurance level has been improved significantly, and has played a role of resources integration in the health service industry. As the core content of the health service industry, health service is influenced by factors such as the scarcity of professional talents, insufficient proportion of the industry, and the limitations of people’s understanding of "service", which result in the lower comprehensive level and slow development of health service.

At the sub-regional level, the comprehensive China’s sports industry and health service industry levels are characterized by the ladder-like spatial distribution of "high in the east and low in the west". In terms of compound annual growth rate, the sports industry is characterized by the distribution of "high in the west and low in the east"; the health service industry is characterized by the highest in the middle, the second in the west, and the lowest in the east. The eastern region belongs to the retarded type of health service industry, and the central and western regions belong to the retarded type of sports industry.

At the provincial level, most provinces and cities where the comprehensive sports industry level has developed well come from the eastern coastal areas, mainly including provinces and cities such as Guangdong, Jiangsu, Shandong, Zhejiang and Beijing. The results of autocorrelation of global space display that the comprehensive China’s sports industry level has a positive aggregation phenomenon in space. Guangdong, Shandong, Sichuan, Zhejiang, Hunan, etc. are the provinces and cities where the comprehensive health service industry level has developed well, and the comprehensive level of each province and city is manifested as random distribution in space. The spatial spillover effect between provinces has yet emerged.

In 2013–2017, the coupling and coordination degree of China’s sports industry and health service industry rose from mild maladjustment to basic coordination, and strode forward the starting stage from the embryonic stage. The coupling and coordination condition of the two industries is also characterized by the ladder-like distribution of "high in the east and low in the west" in space. The regions with better coupling and coordination development are mainly by the eastern coastal provinces and cities as the main bodies, including Guangdong, Shandong, Jiangsu, Zhejiang, Beijing, Sichuan, etc. Guangdong has become the only one region throughout the country at the grade of high-quality coordination.

At the provincial level, the coupling and coordination development of the two industries has gradually changed from "dispersion-type plaques" random distribution in the early phase to the "gathering type scattered surfaces" spatial agglomeration distribution in space. It is concretely manifested as the evolution process of gradient development gradually from the eastern coast to the hinterland with Beijing, the Yangtze River Delta (Zhejiang, Shanghai and Jiangsu) and Guangdong as the three center points and the Changjiang River as the axis.
